# Automated cell-based luminescence assay for profiling antiviral compound activity against enteroviruses

**DOI:** 10.1038/s41598-019-42160-7

**Published:** 2019-04-15

**Authors:** Mingyu Zhang, Yong Zhang, Yan Wang, Wanyu Lv, Yanyang Zhang

**Affiliations:** 1 Immunization Planning Institute, Henan Provincial Center for Disease Control and Prevention, Zhengzhou, Henan People’s Republic of China; 2 WHO WPRO Regional Polio Reference Laboratory, NHC Key Laboratory of biosafety and NHC Key Laboratory of Medical Virology, National Institute for Viral Disease Control and Prevention, Chinese Center for Disease Control and Prevention, Beijing, People’s Republic of China

## Abstract

We describe the development, optimisation, and validation of an automated, cell-based and high-throughput screening assay using existing luminescence-based ATPlite reagents for identifying antiviral compounds that inhibit enterovirus replication. Antiviral efficacy was determined by measuring the ATP levels in cells that were protected from the viral cytopathic effect (CPE) by the antiviral compounds pleconaril and rupintrivir. CPE-based assay conditions were optimised at a cell density of 5000 cells/well and a viral infection dose of 100 CCID_50_ in 384-well plates. The assay exhibited excellent robustness, with Z′-factor values between 0.75 and 0.82, coefficients of variation between 0.33% and 1.45%, and signal-to-background ratios ranging from 6.92 to 22.6 when testing three enterovirus A71 isolates circulating in China. The assay was also suitable for screening other picornaviruses, such as poliovirus, coxsackievirus, echovirus, and parechovirus.

## Introduction

Enteroviruses (EVs; genus *Enterovirus*, family *Picornaviridae*) are small positive-sense RNA viruses that include human enteroviruses, which comprise more than 100 serotypes and are among the most common pathogens that infect humans worldwide, especially children^1^. Most EV infections are asymptomatic, but some may lead to illnesses, ranging from mild to more severe, or even life-threatening, such as hand, foot, and mouth disease (HFMD), aseptic meningitis, encephalitis, myocarditis, pancreatitis, acute flaccid paralysis, and neonatal sepsis^1^. In recent years, outbreaks of HFMD caused by enterovirus A71 (EV-A71) or coxsackievirus A16 have occcured in China and several countries in Southeast Asia^2–6^. To date, there are no approved specific antiviral therapies available to treat diseases caused by EVs. Thus, there is an urgent need to identify safe and broad-spectrum drugs against existing pathogenic EVs.

We selected two well-studied compounds with different viral targets, to aid in the assay optimisation. Pleconaril is an enteroviral capsid inhibitor that inhibits enterovirus replication by blocking virus uncoating and release of viral RNA into cells^7,8^. Pleconaril was recently reported to have anti-enterovirus efficacy in the treatment of neonatal sepsis^9–11^. Rupintrivir (also known as AG-7088) is an irreversible inhibitor of the picornaviral 3 C protease, with demonstrated activity against rhinoviruses and several EVs^12–14^.

To measure cell viability, viral cytopathic effect (CPE) inhibition assays use uptake reagents, such as neutral red, MTT (3-[4,5-dimethylthiazol-2-yl]-2,5-diphenyl tetrazolium bromide), or crystal violet^15^. Limitations of these colorimetric assays include low throughput, requirement for washing steps, low dynamic range, and low signal-to-noise ratio. Cell debris that remains from infected cells can adhere to the microplate, which can result in high background and low signal-to-noise^15^. To overcome these disadvantages, we implemented an alternative assay format using an adenosine triphosphate (ATP) luminescence readout previously developed and validated for a panel of positive-strand RNA viruses^15^. The ATPlite luminescence assay has been used to measure intracellular ATP as a function of cell number and/or viability^16,17^. The advantages of these types of assays are their homogeneous nature, their ability to use both adherent or non-adherent cell lines, and amenability to automation and high-throughput screening (HTS). Our experiments sought to confirm this.

In this study, we describe the optimisation, validation and implementation of an automated cell-based, homogeneous luminescence assay to profile antiviral activity of picornavirus inhibitors against several different EV serotypes, clinical isolates and related picornaviruses.

## Results

### Design of automated HTS assay platform

**Figure 1:**
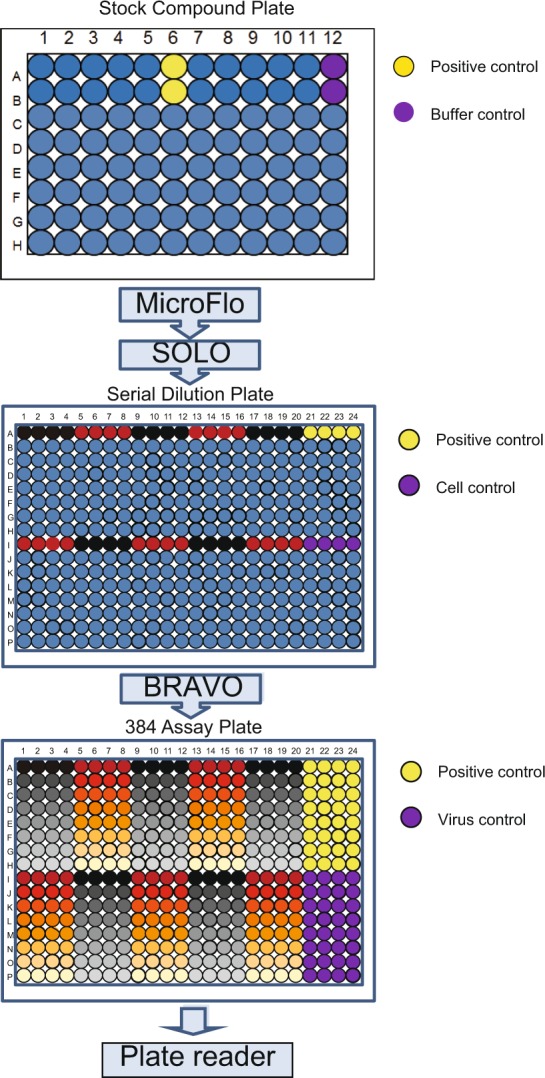
Automated luminescent HTS assay workflow.

We implemented an automated platform (Fig. 1), which took advantage of the homogeneous assay format and ATP/luminescence readout. Small-footprint liquid handlers and reagent dispensers allowed the use of our existing biosafety cabinets. Minimal change to current laboratory space was needed. User-friendly software and custom-written scripts helped the end user in the steep learning curve. A significant component of this platform was the use of the SOLO single-channel robotic workstation and the Bravo Liquid Handling Platform, both of which allowed miniaturisation of the assay from the 300 μL–96-well to the 30 µL–384-well format. This reduced reagent cost and facilitated HTS. Small volume transfers and 384-well serial dilutions were performed with greater accuracy and precision than manual transfers, without the concern of user error (data not shown).

### Assay optimisation

We explored the optimisation of the automated assay (Fig. 1) with acceptable accuracy, precision and sensitivity that would be amenable to a high-throughput setting. Accuracy expresses the closeness of agreement between the value accepted as either a conventional true value or an accepted reference value, and the actual measured value. It is typically measured as percent coefficient of variation (%CV).

The accuracy was determined by replicate analysis of samples containing a known concentration of rupintrivir. Food and Drug Administration guidelines recommend that the %CV should be within +/−15% at all concentrations (http://www.fda.gov/downloads/ucm070107.pdf).

Precision describes the closeness or scatter of individual measures of an analyte obtained for replicate samplings of a homogeneous sample. It is typically measured as %CV. The %CV should be within +/−15% at all concentrations, except for the lower limit of quantitation, where it should not exceed 20% of the CV. (http://www.fda.gov/downloads/ucm070107.pdf).

Sensitivity is defined as the lowest concentration that can be measured with an acceptable limit of accuracy and precision (http://www.fda.gov/downloads/ucm070107.pdf).

### Optimisation of cell density

**Figure 2:**
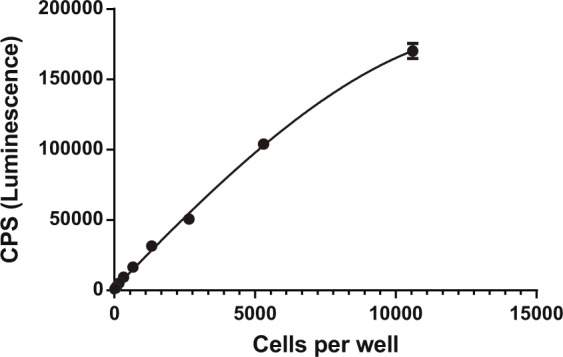
Optimization of RD cell density. Dilutions of RD cells were incubated in 384-well microplates containing MEM with 2% FBS for 72 hours at 37 °C. After incubation, ATPlite reagent was added, and the resulting luminescence was read on a Victor X4 plate reader.

Cell seeding density in 384-well plates was optimised to prevent possible cell overgrowth, which may affect assay sensitivity, and to establish a consistently strong luminescent signal. RD (human rhabdomyosarcoma) cells were seeded in the 384-well microplates at different densities (Fig. 2). The cells were allowed to grow for 72 hours post-seeding, and cell viability was evaluated by adding the ATPlite reagent and reading the luminescence signal. A cell density of 5000 RD cells/well was chosen for the assay because this density gave a consistent and strong luminescence signal, with a good signal-to-background ratio (S/B) ratio and low variability (Fig. 2). Optimal densities for Vero (African green monkey kidney) cells and HeLa (human cervical adenocarcinoma) cells were determined in a similar manner and using the same optimal seeding density.

### Optimisation of virus titre

**Figure 3:**
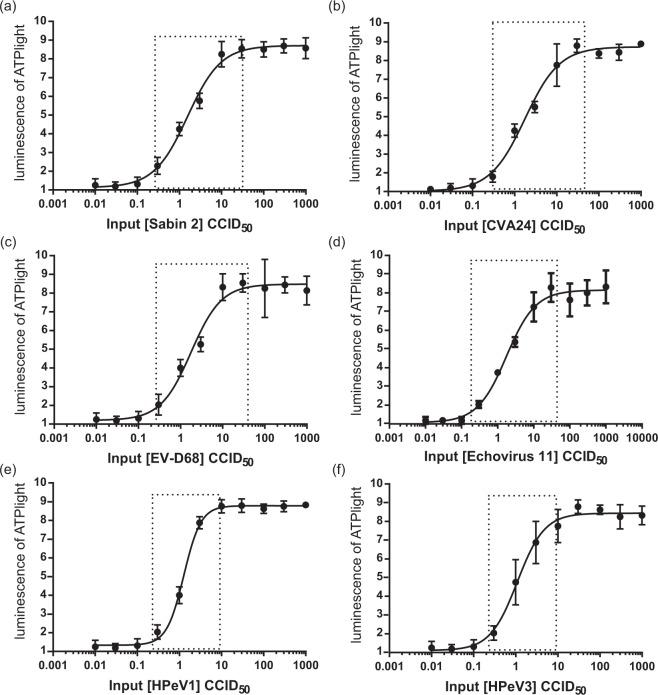
Titration of enteroviruses. HeLa, RD, and Vero cell lines in 50 µL MEM with 2% FBS were inoculated with 50 µL of 0.5-log serial dilutions (in quadruplicate) of viruses and incubated for 72 hours (poliovirus Sabin 2, echovirus 11, and coxsackievirus A24), 120 hours (EV-D68), or 168 hours (HPeV). After incubation, an equal volume of ATPlite reagent was added to determine cell viability (luminescence). The linear range of detection for each virus and cell combination is depicted in the frame and was used to determine the 100 CCID_50_ dilution. (**a**), poliovirus (Sabin2); (**b**), CV-A24 (92183); (**c**), EV-D68 (Ferman); (**d**), Echovirus 11 (US/CA/23901); (**e**), HpeV-1 (Harris); (**f**), HpeV-3 (US/WI/09).

Virus stocks were titrated to determine the dilution that gave optimal sensitivity in the assay. For each virus, the titre was determined by the endpoint dilution method using half-log dilutions in 96-well microplates. A panel of enterovirus and parechovirus strains was established with cell selection and incubation times based on previous experiments^18^. Twelve-point, half-log_10_ serial dilutions of each virus (Fig. 3) were made to determine the starting dilution for use in the automated 384-well assay and to ensure a full dynamic range of the CPE. The starting virus dilutions were selected based on the initial linear portion of the curve for each virus. To induce CPE in at least 80% of the cells, a viral dilution of 100 CCID_50_ was used.

### Validation of automated assay for use in HTS

**Figure 4:**
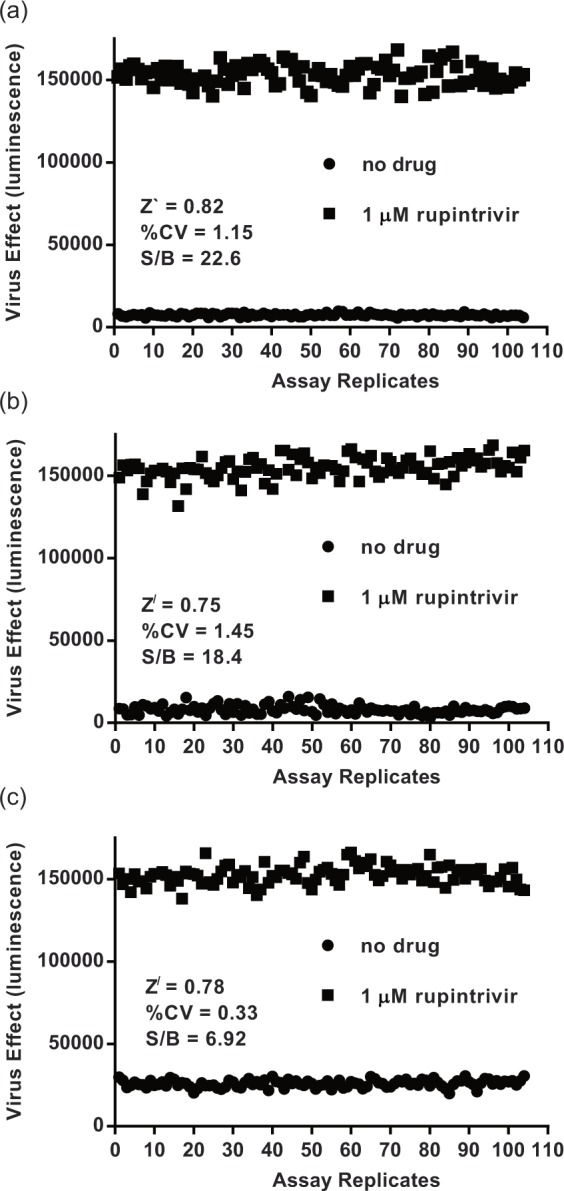
Validation of automated assay for use in high-throughput screening. Determination of the Z′-factor, %CV, signal-to-background (S/B), and EC_50_ were performed on EV-A71 virus isolates from strains circulating in mainland of China. (**a**), strain HuN13-1; (**b**), strain AH-FY-04; and (**c**), strain SH-17. Approximately 5000 RD cells were seeded in 384-well plates and infected with a 100 CCID_50_ dilution of viruses. Half of the plate contained virus and no drug (negative control) while the remainder of the plate contained virus and 1 µM of drug rupintrivir (positive control). After incubation for 72 hours at 37 °C, ATPlite reagent was added, and the resulting luminescence was read on a Victor X4 plate reader.

We evaluated whether the automated assay was suitable for use in a high-throughput setting. The liquid handling instrumentation was configured and the assay robustness was determined using the Z′-factor^19^. The Z′-factor is a coefficient that takes into account the assay signal dynamic range and data variability. Assays with a Z′-factor ≥ 0.5 are considered robust for HTS^19^. During assay development, we defined the optimal assay parameters, including cell density, linear range of detection, Z′-factor [Zʹ = 1 − 3 (SD_DMSO_ + SD_compound_)/|mean_DMSO_ − mean_compound_|], %CV [SD (signal)/average (signal) × 100], and S/B (fold-increase) [signal (cell control)/background (virus control)]. Lower %CV and higher S/B values can contribute to increased assay robustness. Our assay demonstrated robustness, with a high degree of reproducibility, low deviation (including a reproducible Z′-factor between 0.75 and 0.82), and a %CV between 0.33 and 1.45, with S/B ratios ranging from 6.92 to 22.6 (Fig. 4) when using different EV-A71 isolates (Table 1). The additional viruses tested also met these criteria for robustness, with similar results obtained (data not shown).

**Table 1:** *In vitro* activity of pleconaril and rupintrivir against reference viruses.

Cell Line	Serotype-strain	Pleconaril EC_50_ (µM)	Rupintrivir EC_50_ (µM)
HeLa	PV1-Sabin	0.048 ± 0.018	0.221 ± 0.122
HeLa	PV2-Sabin	0.063 ± 0.011	0.289 ± 0.112
HeLa	CV-A24-92183	3.15 ± 0.63	6.52 ± 1.08
HeLa	CV-B3-US/MO/23558	0.345 ± 0.044	0.156 ± 0.024
RD	EV-D68-Fermon	0.36 ± 0.021	0.0028 ± 0.0013
RD	E11- US/CA/23901	0.107 ± 0.016	0.182 ± 0.028
RD	E7- 90201	0.089 ± 0.053	0.017 ± 0.007
RD	EV-A71-HuN13-1	>10	0.332 ± 0.211
RD	EV-A71-AH-FY-04	>10	0.423 ± 0.252
RD	EV-A71-SH-17	>10	0.359 ± 0.281
RD	EV-A71-US/OK/23771	>10	0.022 ± 0.008
Vero	HPeV1- Harris	>10	>10
Vero	HPeV3-US/WI/21001	>10	>10

^a^PV, poliovirus; CVA, coxsackievirus A; CVB, coxsackievirus B; E, echovirus; EV, enterovirus; HPeV, human parechovirus.

### Application of HTS assay for enterovirus susceptibility to known antiviral compounds

**Figure 5:**
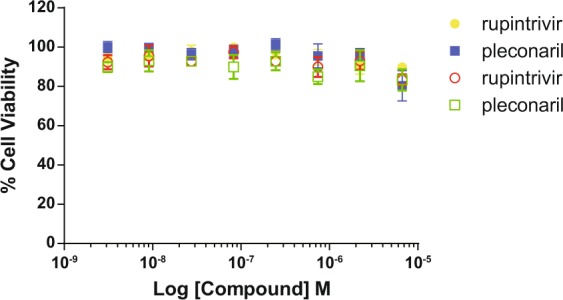
Cytotoxicity of reference compounds. Reference compounds were evaluated in two CPE-based assays. Dilutions of compounds were added to RD cell monolayers in 384-well microplates (luminescent readout, solid symbols) and 96-well microplates (crystal violet readout, open symbols). Assays were incubated for 72 hours at 37 °C. Data represents a mean of four independent experiments.

**Figure 6:**
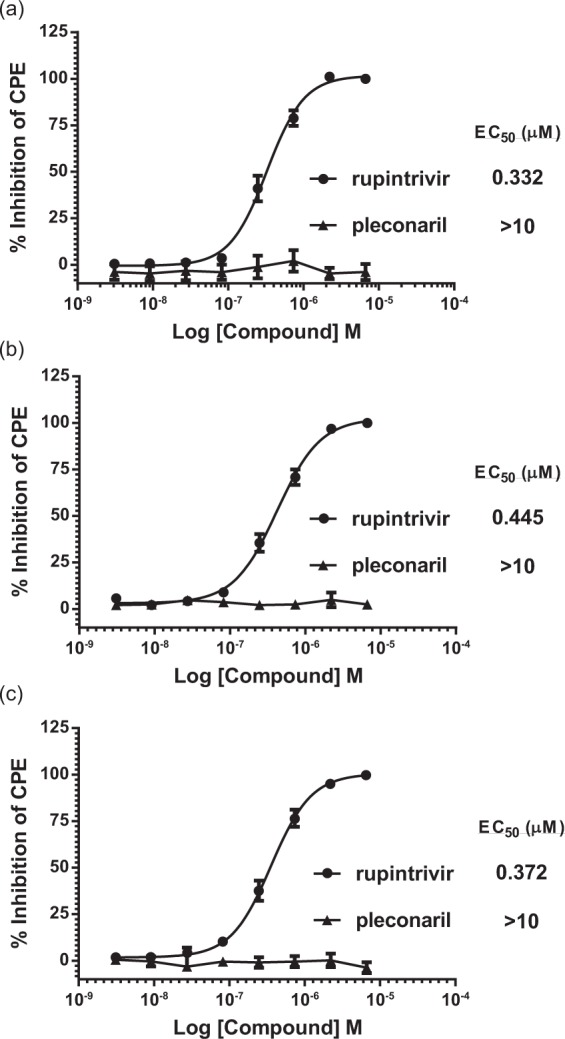
EC_50_ determination of reference compounds. The automated luminescence assay was used to determine the potency of rupintrivir and pleconaril against EV-A71 strains recently circulating in China (strains HuN13-1, AHFY-04 and SH-17). Data represents a mean of four independent experiments. The equation used to calculate the percent inhibition of CPE is: % inhibition of CPE = [(Cells + treated virus) − (Cells + virus)]/[Cells only − (Cells + virus)].

We explored the ability of two documented antiviral compounds (pleconaril and rupintrivir) to inhibit EV isolates and clinical samples (Table 1). Neither compound exhibited cytotoxicity at 10 µM in the automated 384-well luminescent ATP-readout assay or a 96-well assay using a crystal violet readout (Fig. 5). Although a very small subset of EVs was tested, some trends were observed. Pleconaril had more potent 50% effective concentration (EC_50_) values against PV Sabin strains 1 and 2 (0.048 and 0.063 µM, respectively) compared to those of rupintrivir (0.221 and 0.289 µM, respectively). Both compounds had significantly higher antiviral activity against a clinical isolate of coxsackievirus B strain than against a coxsackievirus A strain (0.345 and 0.156 µM compared to 3.15 and 6.52 µM, respectively). Neither compound exhibited antiviral activity against the two types of parechovirus tested. A very pronounced difference was observed when comparing the activities against EV-D68 (strain Fermon). Pleconaril displayed antiviral activity at 0.36 µM, whereas rupintrivir exhibited low, nanomolar antiviral activity of 0.003 µM. This may be due to the targeting of different viral proteins. We tested pleconaril and rupintrivir against four EV-A71 strains. Interestingly, pleconaril had no antiviral activity against any EV-A71 strain (Fig. 6). Rupintrivir exhibited antiviral activity of 0.332, 0.423, and 0.359 µM for three strains circulating in mainland of China (HuN13-1, AH-FY-04 and SH-17, respectively).

## Discussion

Most conventional cell-based antiviral drug screening assays were developed based on cell viability assays (ATPase, kinase activity, and cytotoxicity). The introduction of CPE-based assays using an ATP/luminescence readout of antiviral activity has been recently reported for influenza virus, severe acute respiratory syndrome coronavirus, yellow fever virus, and coxsackievirus^20–23^. This approach measures ATP levels in live cells protected from virus-induced CPE by the presence of antiviral compounds using an ATP-specific luminescent reagent. The advantages of assays with an ATP/luminescence readout are their high-throughput and homogenous (mix and measure) assay format. Homogeneous assays also have the advantage that non-adherent cell lines can be used if desired, and they are amenable to high-density plate formats that can be easily automated. In initial work with RD cells, we observed that these cells did not adhere well to plastic 96-well plates and were easily washed off by our automated system (data not shown). This excluded the use of RD cells in CPE assays requiring adherence of cells prior to staining, which was a distinct disadvantage because RD cells are the optimal cell line for many EVs. In addition, Vero cells do not wash off the plastic cleanly, so reagents like crystal violet stain cell debris that remains adherent, making it difficult to differentiate dead cells from live ones.

Our CPE-based enterovirus antiviral assay used the ATPlite reagent, as the ATP/luminescence readout was easily adapted for use with automation platforms. This assay is also adaptable for use with other cytolytic picornaviruses, using a variety of cell lines in 384-well plates.

Here, an automated, high-throughput, cell-based assay was developed, validated and implemented to profile the efficacy of antiviral compounds against EVs in cell culture. Cell viability measured by ATP detection with a luminescent readout was used as the endpoint. The antiviral compounds pleconaril, a capsid inhibitor, and rupintrivir, a 3C protease inhibitor, were evaluated as model drugs for the inhibition of representative EVs and human parechoviruses. This HTS-suitable assay performed consistently and accurately in an automation-friendly format. This assay is suitable for use to profile other picornavirus inhibitors.

## Methods

### Viruses and cell lines

Polioviruses type 1 and 2 (Sabin vaccine strains) were obtained from the National Institute of Biological Standards and Control (Potters Bar, UK). Coxsackievirus A24 (strain 92183), coxsackievirus B3 (strain US/MO/23558), echovirus 11 (strain US/CA/23901), enterovirus D68 (strain Fermon), and human parechovirus (HPeV) type 1 (strain Harris; formerly echovirus 22) were originally obtained in the 1970s as NIH Reference Reagents (National Institutes of Health, Bethesda, MD). EV-A71 strains HuN13-1, AH-FY-04, and SH-17 were provided by Dr. Wenbo Xu, National Institute for Viral Disease Control and Prevention, China Center for Disease Control and Prevention, Beijing, China. Echovirus (E7) was provided by Dr. Will Weldon, Centers for Disease Control and Prevention, Atlanta, GA.

HeLa (ATCC CCL-2), RD (ATCC CCL-136), and Vero (ATCC CCL-81) cells were maintained in minimal essential medium (MEM; Gibco, Grand Island, NY) with Earle’s salts and 10% fetal bovine serum (FBS).

### Preparation of antiviral compound stock solutions

The capsid inhibitor pleconaril and picornavirus 3 C protease inhibitor rupintrivir were purchased from Sigma-Aldrich (St. Louis, MO). Compounds were stored in 500 µL aliquots at −80 °C, as 2 mM solutions, in high-performance liquid chromatography grade dimethyl sulfoxide (Sigma-Aldrich).

### Automated luminescent antiviral assay

A multichannel pipette was used to transfer 5 µL of 2 mM stock compounds from the stock compound plate (Fig. 1) to rows A and B of a 96-well flat-bottom polystyrene plate (Corning, Corning, NY) containing 245 µL MEM containing 2% FBS (dilution plate). The MicroFlo Select dispenser (BioTek, Winooski, VT), equipped with a rotational wrist and 30-plate stackers, was used to add 60 µL MEM with 2% FBS medium to all wells of a 384-well white flat-bottom plate (Perkin-Elmer, Waltham, MA). The SOLO single-channel robotic workstation (Hudson Robotics Inc., Springfield, NJ) was used to transfer 30 µL of the compound dilutions in quadruplicate into the 384-well serial dilution plates (Fig. 1). The Bravo Liquid Handling Platform (Agilent Technologies, Inc., Santa Clara, CA) executed 30 µL half-log_10_ serial dilutions in the serial dilution plate and then transferred 5 µL of the diluted compounds into multiple assay plates (Fig. 1). Cells were diluted to 2.5 × 10^5^ cells/mL in MEM with 2% FBS, and 20 µL were added to each well of the assay plates using the MicroFlo Select reagent dispenser. The assay plates containing compound dilutions and cells were incubated for 1 hour at room temperature prior to adding 100 CCID_50_ dilutions of viruses. The MicroFlo Select reagent dispenser was used to dispense 5 µL of virus in MEM (Gibco) with 2% FBS (HyClone, Thermo Fisher Scientific, Waltham, MA) to 384-well assay plates. Viruses were added to the entire plate, including virus control wells that did not contain test compound. The assay plates were covered with a plate lid, wrapped in plastic wrap with a wet paper towel placed at the bottom of the stack and incubated at 37 °C in a 5% CO_2_ atmosphere for 3–7 days, depending on the virus and cell line^18,24^. The final assay volume was 30 µL.

After the appropriate incubation period of the cell-virus combination, 15 µL of cell lysis buffer followed by 15 µL of reconstituted substrate solution were added to each well using the MicroFlo Select reagent dispenser. After incubation for 10 minutes in the dark, the resulting luminescence was read on a Victor X4 plate reader with a 0.1 second integration for each well. Raw luminescence counts were exported in Excel software (Microsoft, Redmond, WA) to custom spreadsheets that calculated the percent inhibition of CPE in each well. The definition of the percent inhibition of CPE was calculated as:

$${\rm{Percent}}\,{\rm{inhibition}}\,{\rm{of}}\,{\rm{CPE}}=[({\rm{Cells}}+{\rm{treatedvirus}})-({\rm{Cells}}+{\rm{virus}})]/[{\rm{Cells}}\,{\rm{only}}-({\rm{Cells}}+{\rm{virus}})]$$

### Colorimetric antiviral assay

An *in vitro* CPE assay (signal-to-noise ratio >5) was used to calculate the EC_50_, expressed in µM, for a given virus isolate. Compound and virus were combined with cells (2 × 10^5^ cells/well) in 96-well plates, starting at 10 µM of the compound. To ensure that endpoints from compound and virus titrations were reached, 11 seven-point titration curves were performed in 0.5 log_10_ steps with duplicate wells for each compound-virus concentration. Three replicate tests were run for each virus, unless otherwise stated. After appropriate incubation at 37 °C, cells were stained with crystal violet (0.05% crystal violet, 0.5% Tween-20 and 50% ethanol in deionized water) and washed three times with deionized water. Plates were air-dried and viral CPE was measured by determining the absorbance at 590 nm.

### Data analyses

Data were analysed by a four-parameter curve-fitting using GraphPad Prism (version 5.0.3, GraphPad Software, LaJolla, CA) to calculate the EC_50_ of each compound. A minimum of five tests were run on each virus-compound combination. If the results of the five tests fell within one half-log_10_ drug dilution, the mean EC_50_ value with the standard deviation of the mean was reported. If the results were not within one drug dilution, another five tests were conducted, and the results were averaged. If the virus dilution did not achieve the desired >80% destruction of cells, the virus was passaged to increase the titre. If the virus was inhibited at all compound concentrations, the test was repeated starting at a 10-fold lower concentration of the compound.

## Data Availability

The data used to support the findings of this study are included within the article.
